# Education Research: Guided Worksheets to Support Residents as Clinical Teachers in the Neurology Clerkship

**DOI:** 10.1212/NE9.0000000000200311

**Published:** 2026-05-13

**Authors:** Clare McGarvey Lambert, Jeffrey Dewey

**Affiliations:** 1 Department of Neurology, Johns Hopkins Hospital, Baltimore, MD; and; 2 Department of Neurology, Yale School of Medicine, New Haven, CT.

## Abstract

**Introduction and Problem Statement:**

Neurology residents are often tasked with incorporating impromptu medical student teaching into their clinical workflow, yet few resources exist that combine content and pedagogical guidance in an efficient format. Residents are also learning themselves and may experience anxiety around teaching, compounded by the additional cognitive load of identifying and addressing student learning needs. We developed a guided worksheet tool to make high-yield, effective clinical neurology teaching accessible to busy residents.

**Objectives:**

We piloted 4 self-guided worksheets on core neurology topics and examined student learning and the resident teaching experience. We aimed to (1) demonstrate successful student knowledge acquisition, retention, and satisfaction and (2) demonstrate improved resident teacher (RT) comfort, efficacy, and/or efficiency in clinical teaching on inpatient services.

**Methods and Curriculum Design:**

Four worksheets were developed: ischemic stroke basics, reading magnetic resonance imaging of the brain, intracranial hemorrhage, and seizures. Residents were asked to guide students through filling out a partially blank version, using a completed ‘master’ version as a guide, while providing targeted teaching when knowledge gaps were apparent. Student learning was assessed using presession, postsession, and two-week postsession quizzes. Qualitative analysis was performed on semistructured interviews with RTs to understand their experience teaching with the tool.

**Results and Assessment Data:**

Six RTs taught 27 medical students a total of 47 lessons. Learner knowledge scores improved immediately after lessons (1.24/3 ± 0.92 before lesson vs 2.75/3 ± 0.49 after lesson, *p* < 0.001) and were sustained at 2 weeks after lesson (1.24/3 ± 0.92 before lesson vs 2.47 ± 0.61 2 weeks after lesson, *p* < 0.001). RTs believed that the tool improved their clinical teaching, with themes of reducing stress around impromptu teaching, improving basic content delivery, the power of microlearning, and developing self-efficacy identified in qualitative interviews.

**Discussion and Lessons Learned:**

Guided worksheets offer content and can support RTs in providing effective clinical teaching while improving their comfort and self-efficacy. Expansion of these tools to additional high-yield topics may offer further support for resident teaching in the clinical setting.

## Introduction and Problem Statement

As medical school enrollment rises, class sizes grow, and protected time for faculty to teach declines, residents are becoming increasingly integral in their role as teachers during Neurology rotations while simultaneously juggling the demands of higher patient loads and case complexity on inpatient services.^1,2^ Residents are thus in a unique—and potentially anxiety-provoking—position of teaching topics they may not yet have mastered themselves. Trainees frequently identify teaching as an important part of their job but also endorse feeling overwhelmed by clinical duties and unprepared to be a teacher.^3-5^ Many popular structures for learner-centered clinical teaching (e.g., One Minute Preceptor, SNAPPS, and SPICES) exist but provide only procedural guidance.^6-10^ Content guidance for neurology clerkship teachers is available,^11^ but distilling high-yield learning points from a field that students already find overly complex can be prohibitively challenging for residents who have yet to clarify their own schemas.^12,13^

We hypothesized that developing a simple, user-friendly education tool that focuses on approachable basic frameworks could result in effective transmission of high-yield learning points. Fill-in-the-blank worksheets have been effective in other subspecialties but, to our knowledge, have not yet been studied in Neurology.^14-16^ We, therefore, designed guided worksheets that can be easily deployed off-the-shelf on an inpatient neurology service and examined whether these could aid residents in teaching core content while enhancing resident comfort and self-efficacy in impromptu clinical teaching.

## Objectives

We piloted 4 self-guided worksheets on core neurology topics and examined student learning and the resident teaching experience. Our objectives were to (1) demonstrate student satisfaction and learning efficacy (i.e., Kirkpatrick levels 1 and 2) and (2) assess the impact of this teaching tool on the resident experience of teaching in a busy clinical environment.

## Methods and Curriculum Design

### Guided Worksheet Development and Topic Selection

We defined a “guided worksheet” as a premade framework for teaching a core component of the Neurology rotation that uses a mix of prefilled areas and blank spaces for learners to fill in as the lesson progresses. RTs can use this framework to guide students through the topic, using a completed answer key as a reference if needed (eFigures 1–4). We developed 4 guided worksheets that covered topics commonly requested by medical students that aligned with the Yale School of Medicine core neurology clerkship content: (1) ischemic stroke basics, (2) intracranial hemorrhage (ICH), (3) reading brain MRI, and (4) seizures. Content was developed from clinical literature, local expert opinion, and American Academy of Neurology Continuum resources.^17-22^ Worksheets were created by a PGY-4 academic chief resident (C.M.L.) and reviewed by the neurology Clerkship Director (J.D.) to ensure content accuracy and alignment.

### Teaching Instructions

Teachers were instructed to allow the student to select the teaching topic and lead the pace/direction of the session. Educators were told that they did not need to prepare beforehand, as the tool was intended to be flexible and meet the needs of the learner. Teachers were encouraged to give learners the opportunity to try to fill out the blank spaces of the worksheet on their own and only help them when they were unable to do so. It was recommended that teachers encourage questions from learners and use the questions to guide the flow and structure of the lesson. Teachers were free to only focus on part of the guided worksheet or complete it in its entirety. Students were allowed to keep the completed worksheet at the end of the lesson for reference and further individual learning.

### Resident Teacher Recruitment

Paper copies of the guided worksheet and answer keys were made available in the resident workrooms. All senior residents were informed of the study and worksheet availability by email and a presentation at the monthly resident meeting. Resident participation was voluntary. C.M. Lambert was available at any time to answer questions and further guide RTs. When residents chose to use a worksheet during teaching, they were encouraged to offer the opportunity to participate to any available students; therefore, some sessions could include students from the general inpatient, neuroscience intensive care unit, stroke, consult, and pediatric services together, leading to groups of 1–5 students present per session. Lessons were not prescheduled, and lesson duration was determined by student learning needs and engagement. Lesson topic was chosen by mutual agreement between the RT and student(s). No compensation for participation was provided.

### Student Recruitment and Data Collection

RTs offered the opportunity to participate in data collection to all students, although participation in the teaching lesson was not contingent on study consent. If the students consented to study participation through a quick response (QR) code, they were then directed to a prelesson quiz through a second QR code. Participating students (generally MS2 and MS3 based on our institution's curricular structure) were asked to complete a postlesson quiz and survey, including the option to provide their email address if they were willing to complete a two-week postlesson quiz for assessment of learning retention. All quizzes were linked to an anonymous, unique identifier generated by the student. Data were stored anonymously in Qualtrics. No participant demographic information was collected.

### Student Satisfaction Assessment

After completion of the postlesson quiz, participating students were prompted to complete a five-question Likert-scale satisfaction survey, which was recorded in Qualtrics anonymously. Students were asked to select “agree,” “neutral,” or “disagree” with 5 statements: (1) I found this tool effective, (2) I enjoyed filling out the scaffold, (3) this was an efficient way to learn this topic, (4) I am likely to refer back to this scaffold in the future, and (5) I would like to learn other topics in this format in the future. “Agree” was assigned 1 point, “neutral” 0 points, and “disagree” -1 point where a sum of 5 would reflect agreement with all statements indicating the highest level of satisfaction, 0 would reflect a neutral response to all statements, and -5 would reflect disagree with all statements indicating the lowest level of satisfaction.

### Student Learning Assessment Development and Score Analysis

The knowledge quizzes were developed by the authors based on the content of the guided worksheet (eAppendixes 2–5). Quizzes were limited to 3 questions to favor completion during busy services. Prelesson (t_1_) and immediate postlesson (t_2_) scores, as well as prelesson and 2-week postlesson (t_3_) scores, were compared across the total group as well as the 4 lesson subgroups using the Wilcoxon signed rank test, because of the non-normal distribution of post-test scores and pre/post data structure. Alpha (α) was set to 0.05 for exploratory t_1_ vs t_2_ and t_1_ vs t_3_ subgroup comparisons. Bonferroni-corrected alpha (α’ = $\frac{\alpha }{k}$ = 0.017) was used where multiple comparisons occurred. Data analysis was performed in R.^23,24^

### Post-teaching Interviews and Qualitative Analysis

Any residents who delivered at least one lesson over the course of the study were given the opportunity to participate in a semistructured interview conducted by an education chief resident (C.M. Lambert). Interviews were conducted over the phone in a private area during a prescheduled session. The interviewer asked 4 core questions in each interview, with clarifying questions as needed to ensure understanding and completeness of answers: (1) Before using the scaffolds, how did you feel about giving medical students unstructured or off-the-cuff “chalk talks”?; (2) How does teaching fit into a day on inpatient service for you?; (3) Did you find having the scaffolded templates useful? If yes, in what way?; and (4) What changes would you make to the scaffolds in the future and what other topics would you find useful to have covered this way? Interviews were transcribed, deidentified, and stored using the NVivo software platform.^25^ Each transcript was coded separately by both study authors and subsequently reviewed iteratively for consensus. No new themes emerged between the fourth and fifth interviews. Given both authors' involvement in worksheet development, specific consideration was given to potential bias during thematic analysis, although we acknowledged the advantage of having deep contextual familiarity when conducting interviews and analysis. In addition, C.M. Lambert, as an education chief resident, had an established track record of receiving feedback from co-residents regarding various educational initiatives in the residency.

### Standard Protocol Approvals and Registration

The study was approved by the Yale University Institutional Review Board and was deemed exempt (study 2000036183). The study and student consent forms were approved by the Yale School of Medicine Committee to Review Student Participation in Research. This study was not funded. The STROBE checklist for observational research was followed.^26^

### Data Availability

Anonymized data not published within this article can be made available by request from any qualified investigator.

## Results and Assessment Data

Worksheets were deployed during Neurology clerkships from 2023 to 2025, and 6 of the 24 available Neurology and Child Neurology residents (PGYs 3–5, including C.M.L.) used this tool over the course of the study, delivering 47 unique lessons to 27 medical students. Five RTs, excluding C.M. Lambert, were interviewed. Emergent codes/subthemes were later organized into the categories/parent themes of (1) teaching experience, (2) the tool in practice, and (3) tool feedback. Themes, subthemes, and representative quotes are listed in Table 1.

**Table 1:** Themes and Illustrative Quotes From Resident Interviews

Parent theme and definition	Subtheme	Illustrative quotes
Teaching experience *Residents' description of what it is like being a teacher*	Challenges facing resident teachers	“I don't feel comfortable. I always have to… plan out a lecture if I'm going to do a lecture. If I'm put [on the spot] to give a chalk talk there are very little topics that I'm actually comfortable [teaching]” “…I'm talking about a topic and then sometimes I'll remember that there was another concept that I should have brought up during the talk later” “Prior to using the scaffold it was difficult to know what each student's baseline was… like, how in depth do I go?”
	Challenges around making time for teaching	“Unfortunately, depending on the rotation requirements, a lot of teaching is going to be informal teaching… in the afternoons is when I try to do some more formalized teaching [but] there is only a couple of days that could work for.”
The tool in practice *How the tool performed in real life and how residents grew as teachers after using the tool*	Using the tool flexibly	“Even if I don't get around to explaining every single concept, they can just hold on to the sheets for self-learning, so it has been helpful in that sense.” “It's easier to digress and make interesting points when you have the scaffold there.”
	Teaching as a means of learning	“Having a helpful guide to teach off of, it would be helpful and solidify my own knowledge.” “It was good for … reminding myself [of] those specific points that I might have forgotten.”
	Developing self-efficacy	“I've given a version of that [scaffold] so many times now that I don't need the scaffold in front of me to be able to go through it all.” “Students could, starting their second or third day, readily apply the TOAST criteria and use that for rounds” “First time seizure… is definitely a topic that for me was easy to be a little bit all over the place in terms of talking about the different presentations and what you look for. And [the scaffold] helped me be much more systematic about it.”
	Tailoring to the learner	“I think it's helpful in that it provides me with an outline that I can then fill in with knowledge targeted at the student's knowledge gaps.” “For students who have a greater fund of knowledge, the scaffold is just enough information and structure for me to be able to then ad lib a lot of teaching. For people who are less well read in their neurology it serves as a good background into the topic.”
	Understanding the power of microlearning	“There are some cases where I've had students say ‘oh, I wish I could learn about neuroimaging’, but it's on a very busy service day in the NICU, and even then I was able to just give them the worksheet.” “If we have some time before lunch, then it's actually pretty helpful to go over the stroke buckets.” “I had an extra 10 min before rounds started and I was like, let's quickly go through this [stroke worksheet]… you can be focused, efficient, you don't forget about it and it doesn't get swept under the reg and you can actually accomplish a lot in a little window… like a bite sized nugget.”
Tool feedback *Residents' feelings about the scaffolded worksheet.*	Covering the basics	“It's a good structure to teach med students the basic concepts.”
	Engaging	“I think it allowed the med students to be kind of engaged and have a structure in which they can take notes and learn.”
	Expanding the scope/future directions	“There's so many [topics] that could be helpful.” “[The students] could work on it asynchronously… and give the answer key to the students.”
	Sense of security	“I mean it gave me some security that it was a structure that was used before and I could just abide by it and sort of add on my [own] clinical knowledge”
	Need for better accessibility	“Try to get people to be aware [of the scaffold's] existence.” “I think they're probably under-utilized.”
	Constructive criticism	“…just the [brain bleed] timing one… writing [‘I bleed’, ‘I die’, ‘Bleed, die’, ‘Bleed, bleed’, ‘Die, die’] as a mnemonic for the timeline, but I think explaining the rationale behind hyperacute MRI would be [more] helpful.” “I don't know if information could have been better just given to them…like advanced stroke workup, just so they're aware of it instead of having it be [blank], because we might not always get there. I think focusing the fill-in-the-blanks on the more simple concepts…leaves you more room for discussing [higher-yield] concepts.”

### Teaching Experience

Residents tended to focus on challenges they faced when describing their experience with impromptu teaching. RTs noted initial feelings of anxiety, fear of being disorganized, and concern about leaving out important information when challenged to deliver spontaneous teaching to medical students before using the guided worksheets. For example, one resident described how they might scribble down some free-hand notes on the back of a patient list, and students may snap a picture of this framework or ask to keep the notes afterward, which led to a heightened sense of disorganization and anxiety around the teaching experience. Another resident described a feeling of “chaos” during teaching encounters due to jumping around from idea to idea without a structure in mind. Time pressure limited high-quality teaching, and it was not always clear to residents what topics they should teach or at what depth.

### The Tool in Practice

RTs who regularly used the worksheets were able to teach flexibly within the template and adjust to different learners' knowledge gaps. Some residents reported subsequent comfort teaching that topic without organizational aids. Residents felt that teaching with the worksheets also reaffirmed their own basic Neurology knowledge. Finally, residents began to realize the power of microlearning, that is, that brief impactful lessons can be delivered on a busy service.

### Tool Feedback

Overall, residents found the guided worksheets helpful. They appreciated the security of their basic structure and reported student engagement. Residents identified a lack of awareness of this tool as a barrier to its broader use. When prompted for constructive feedback, residents noted that there were some areas where more prefilled content could improve delivery (e.g., advanced stroke workup) and some components may have overemphasized rote memorization (e.g., aging blood on MRI using a mnemonic). They identified multiple sclerosis, peripheral anatomy (upper and lower limb), ventilator management in the neurosciences intensive care unit, subarachnoid hemorrhage management, differential diagnosis of headache, and Parkinson disease/Parkinson plus syndromes as high-yield future topics.

### Student Satisfaction and Learning

Mean student satisfaction scores were 4.49/5. Participating students (n = 27) completed 46 t_1_, 41 t_2_, and 19 t_3_ quizzes (14 ischemic stroke, 9 seizure, 13 MRI, and 10 ICH). Score data are provided in Table 2. Overall, scores improved significantly between t_1_ and t_2_ assessments (n = 41 pairs, V = 666, *p*_α≤0.017_ < 0.001). Improvement was sustained when comparting t_1_ and t_3_ scores (Table 2; n = 19 pairs, V = 165, *p*_α≤0.017_ < 0.001). However, when t_2_ and t_3_ scores were compared, knowledge scores had a small but statistically significant drop (n = 19 pairs, V = 0, *p*_α≤0.017_ = 0.011).

**Table 2:** Prelesson, Postlesson, and 2-Week Postlesson Knowledge Quiz Scores

Lesson	Time point	n	Mean ± SD	Median	*p* Value
Stroke	Prelesson	14	0.25 ± 0.50	0.00	—
	Postlesson	12	2.75 ± 0.25	3.00	**0.005**
	2-Week postlesson	4	2.25 ± 0.96	2.50	0.100
Seizure	Prelesson	9	1.50 ± 1.29	1.50	—
	Postlesson	8	2.75 ± 0.50	3.00	**0.008**
	2-Week postlesson	4	2.25 ± 0.50	2.00	0.345
MRI	Prelesson	13	1.86 ± 0.69	2.00	—
	Postlesson	12	3.00 ± 0.00	3.00	**0.005**
	2-Week postlesson	7	3.00 ± 0.00	3.00	**0.031**
Brain bleed	Prelesson	10	0.50 ± 0.58	0.50	—
	Postlesson	8	2.75 ± 0.50	3.00	**0.021**
	2-Week postlesson	4	1.75 ± 0.50	2.00	0.090
Total	Prelesson	46	1.24 ± 0.92	1.00	—
	Postlesson	40	2.75 ± 0.49	3.00	**<0.001** ^a^
	2-Week postlesson	19	2.47 ± 0.61	3.00	**<0.001** ^a^

The mean, standard deviation, and median of the prelesson, postlesson, and 2-wk postlesson knowledge scores are displayed. Quizzes consisted of 3 questions, each worth one point.

a All comparisons use the Wilcoxon rank-sum test and the prelesson score serves as the comparator group, *α* = 0.05. Significant *p* values are given in bold.

Bonferroni-corrected *α* = 0.017 was used for total group comparisons where multiple comparisons took place (t_1_ vs t_2_, t_1_ vs t_3_ and t_2_ vs t_3_; in Results section).

## Discussion and Lessons Learned

Our findings highlight one approach to pragmatically bridge the gap between medical education theory and practice for busy neurology residents. Guided worksheet lessons proved effective for student learning and alleviated initial discomfort with impromptu clinical teaching that our residents share with other studied groups of RTs.^27,28^ In addition, the worksheets prompted learner-centered teaching by modeling inquiry-directed teaching targeting knowledge gaps that were identified in real time, which improved resident confidence in impromptu clinical teaching and affected subsequent teaching behaviors. Although not directly compared with a control, worksheets met the baseline expectation of being satisfactory and effective for student learning (Kirkpatrick levels 1 and 2).^29^

Based on these positive initial findings, we plan to continue building lesson worksheets to cover a broader range of core neurology content and incorporating further RT feedback to hone these tools. As with many educational interventions, however, uptake remains the greatest roadblock. Although the residents who chose to use our worksheets found them effective, we observed relatively low utilization over the course of the study. This may be related to focusing recruitment on senior residents in our program because they most often perform medical student–directed inpatient clinical teaching. However, many senior residents have already developed their own teaching repertoire and thus may not have felt a need for new tools. We polled junior residents on their likelihood of using this tool and their feelings around providing impromptu teaching during our early study design (in eAppendix 1 and eTable 1), but a true comprehensive needs assessment that better characterized the learner and teacher experience before designing our tool would have been informative, as would extending use of the tool to more junior teachers in the future. Finally, we did not assess why RTs chose to use the tool or interview those who chose not to, so it is possible that the residents who were most excited about medical education, or those least confident in their teaching abilities, self-selected to participate.

We also acknowledge methodological limitations to these conclusions. First, the script used to guide the semistructured interview was designed to be concise but informative and exploratory; however, it was not piloted beforehand, and as such, we cannot be certain that phrases such as “off-the-cuff chalk-talk” and “scaffolded worksheets” were understood by all RTs; that said, no clarification of these phrases was requested during interviews. Of note, we use the term “scaffold” in interviews to describe our active learning too but did not explicitly track the degree to which students relied on their worksheets for knowledge/organization as their rotation progressed. Second, we opted not to record session length or the degree of worksheet completion, as both were intended to be flexible based on learner needs, but these data could have yielded some additional insight into worksheet appropriateness. Finally, we only offered 4 core Neurology topics in this pilot, which may have hindered our teacher's ability to address other student learning gaps. However, these topics were deliberately chosen to leverage topics that are foundational and often requested by students on inpatient rotations.

Finally, we recognize several limitations to our student assessment. Our knowledge quizzes were not validated and were intentionally brief for ease of use but thus did not capture the depth of information covered in each lesson. Conversely, the flexible nature of the guided worksheets may have resulted in incomplete coverage of the information tested in the three-question quiz, despite execution of an effective lesson. There also may have been a priming effect, in which students were biased by the prelesson quiz and, therefore, pushed the lesson toward focusing on the question content. Finally, some of the question answers have overlapping components, which is not optimal practice during test item development.^30^ In the future, we would also like to interview students who engaged with the tool, as they are key stakeholders.

Our early positive findings suggest numerous potential future directions. First, in the spirit of learner-centered teaching, a larger library of worksheet lessons is necessary to cover a wider range of potential student needs, ideally aligned with the American Academy of Neurology's curriculum guidelines.^11^ More extensive evaluation of these guided worksheets in larger student cohorts, among teachers with varying levels of experience, and potentially across multiple institutions would yield more nuanced data and drive further curricular refinement. Indeed, faculty stand to reap the advantages of these tools as well, particularly those who do not regularly engage in clinical teaching and desire guidance on what content should be covered during inpatient neurology rotations. Further longitudinal follow-up interviews can elucidate whether residents have changed teaching practices to integrate these guided worksheets (Kirkpatrick level 3).^29^ More broadly, we hope to eventually learn whether increasing accessibility of clinical neurology content will meaningfully improve student engagement.

In conclusion, our findings highlight one method for empowering educators to effectively engage students in busy clinical environments and build confidence as teachers. This proof-of-concept study lays the groundwork for broader development and validation of these tools to nurture neurology's greatest resource: its passionate clinical teachers.

## Glossary

ICH: intracranial hemorrhage
RT: resident teacher
